# Characteristics of ^18^F-FDG and ^18^F-FDOPA PET in an 8-year-old neutered male Yorkshire Terrier dog with glioma: long-term chemotherapy using hydroxyurea plus imatinib with prednisolone and immunoreactivity for PDGFR-β and LAT1

**DOI:** 10.1080/01652176.2021.1906466

**Published:** 2021-05-05

**Authors:** Taesik Yun, Yoonhoi Koo, Sanggu Kim, Wonguk Lee, Hakhyun Kim, Dongwoo Chang, Soochong Kim, Mhan-Pyo Yang, Byeong-Teck Kang

**Affiliations:** a Laboratory of Veterinary Internal Medicine, College of Veterinary Medicine, Chungbuk National University, Cheongju, Chungbuk, South Korea; b Department of Veterinary Medicine, College of Veterinary Medicine, Chungbuk National University, Cheongju, Chungbuk, South Korea; c Department of Nuclear Medicine, Chungbuk National University Hospital, Cheongju, Chungbuk, South Korea

**Keywords:** Dog, canine, 18F-FDG, 18F-FDOPA, glioma, hydroxyurea, imatinib, L-type amino acid transporter 1, positron emission tomography

## Abstract

An 8-year-old neutered male Yorkshire Terrier dog presented with head pressing, vestibular ataxia, neck tenderness, and no oculocephalic reflex. A demarcated lesion in the pons was identified on MRI. The patient was tentatively diagnosed with a glioma and was treated with hydroxyurea plus imatinib and prednisolone. After 30 days of therapeutic treatment, the patient showed a clear improvement in neurological signs, which lasted for 1117 days. On day 569 after the initiation of treatment, ^18^F-fluorodeoxyglucose (FDG)-positron emission tomography (PET) was performed with no significant findings on visual analysis. The average and maximal standardized uptake values (SUVs) were 1.92 and 2.29, respectively. The tumor-to-normal-tissue (T/N) ratio was 0.97. The first evidence of clinical deterioration was noticed on day 1147. On day 1155, 3,4-dihydroxy-6-[^18^F]-fluoro-l-phenylalanine (^18^F-FDOPA)-PET was performed. High uptake of ^18^F-FDOPA was observed in the intracranial lesion. The mean and maximal SUVs of the tumor were 1.59 and 2.29, respectively. The T/N ratio was 2.22. The patient was euthanized on day 1155 and histopathologic evaluations confirmed glioma (astrocytoma). This case shows that chemotherapy with hydroxyurea plus imatinib may be considered in the treatment of canine glioma. Furthermore, this is the first case describing the application of ^18^F-FDG and ^18^F-FDOPA in a dog with glioma.

An 8-year-old neutered male Yorkshire Terrier dog presented with a history of head pressing, and an episode of vestibular ataxia was observed 2 days prior to presentation. On physical examination, the patient weighed 5.3 kg, had a pulse rate of 116 beats per minute, a respiratory rate of 48 breaths per minute, and a rectal temperature of 38.9 °C. On neurological examination, the patient exhibited neck pain, and the oculocephalic reflex was not elicited. Biochemical analysis did not show any remarkable abnormalities except for mildly increased alkaline phosphatase activity. Based on the clinical signs and findings on neurological examination, the lesion was neuroanatomically localized to the brainstem.

MRI of the brain was performed using a 0.3-Tesla unit (Airis II, Hitachi, Japan). General anesthesia was induced with intravenous administration of propofol (6 mg/kg BW; Provive, Myungmoon Pharm. Co., Ltd, Seoul, South Korea) and maintained by inhalation of 2.0–2.5% isoflurane (Terrell, Piramal Critical Care, Bethlehem, PA, USA) in 100% oxygen in a circle rebreathing circuit. T1-weighted (pre- and post-contrast), T2-weighted, and fluid-attenuated inversion recovery (FLAIR) images were obtained using transverse, sagittal, and dorsal planes. The lesion was identified in the pons, and peritumoral edema was found around the lesion. Hyperintensity in T2-weighted (Figures 1A and D) and FLAIR (Figure 1B) images and hypointensity to isointensity in T1-weighted images were observed. After administration of gadolinium-diethylenetriamine pentaacetic acid [0.1 mmol/kg BW, IV; Omniscan^TM^, GE Healthcare (Shanghai), Co., Ltd, China], post-contrast images were acquired, and ring enhancement was noted (Figure 1C). Cerebrospinal fluid cytology was not performed owing to blood contamination during tapping; however, polymerase chain reaction was negative for the following infectious agents: *Bartonella spp., Blastomyces dermatitidis, Coccidioides spp., Cryptococcus spp., Histoplasma capsulatum,* Canine distemper virus, West Nile virus, *Borrelia burgdorferi, Neospora spp.,* and *Toxoplasma gondii*. Based on history, signalment, clinical assessments, and MRI features (intra-axial origin mass and ring enhancing pattern without dural tail sign), a glioma was strongly suspected.

**Figure 1.**
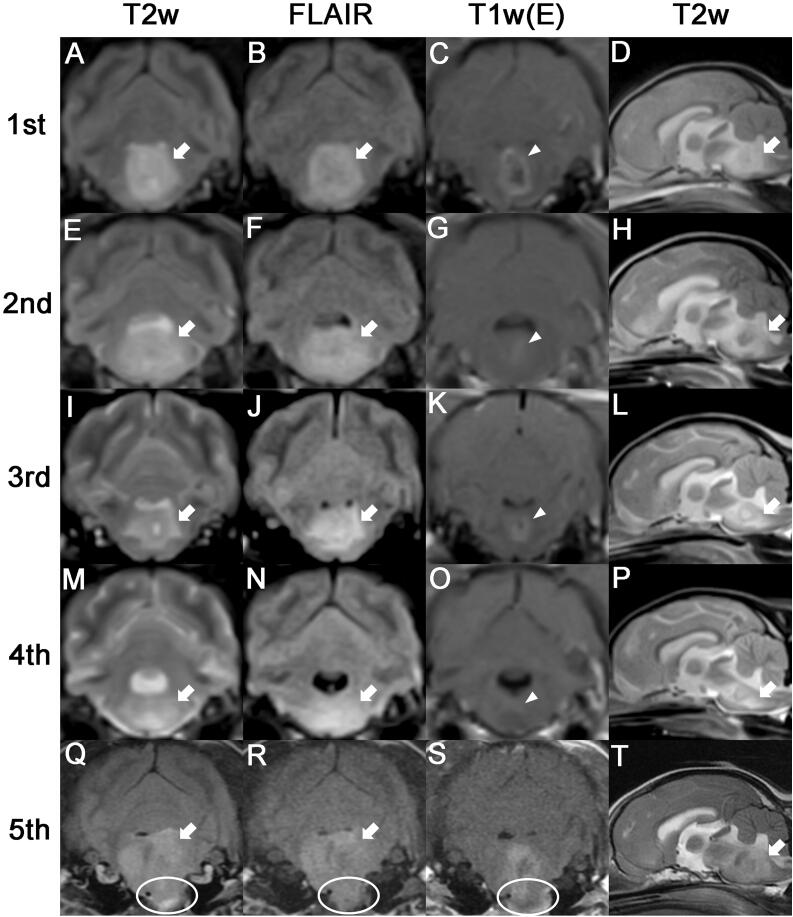
Serial MRI characteristics of astrocytoma in an 8-year-old neutered male Yorkshire Terrier dog. The first four MRIs were performed using a 0.3-Tesla unit, and the fifth scan was performed using a 1.5-Tesla unit. (A-D) The first MRI scan was acquired before chemotherapy. The tumor lesion (arrows) showed hyperintensity on T2-weighted and fluid-attenuated inversion recovery images. Postcontrast image exhibited ring enhancement (arrowhead). (E-H)Second MRI taken 30 days after chemotherapy. A larger area of peritumoral edema (arrows) compared with that observed on the first MRI scan was observed, and a reduction in the size of the contrast-enhanced lesion (arrowhead) was observed. (I-L) Third MRI scan (taken on day 213). The peritumoral edema (arrows) decreased compared with previous MRI observations, and the contrast enhanced mass (arrowhead) slightly increased in size. (M-P) Fourth MRI scan (taken on day 569). While the peritumoral edema (arrows) increased to a slight extent compared with the observation from the third MRI scan, contrast enhancement (arrowhead) was rarely observed. (Q-T)Fifth MRI scan (taken on day 1155). A much larger tumor (arrows) than those observed on previous MRI scans was noted, and protrusion of the tumor (circles) into the nasopharynx was also observed.

For metastatic evaluation, the patient underwent radiography and ultrasonography on the day after the MRI scan, and no metastatic lesion was observed.

At the owner’s request, surgery was not performed owing to its non-peripheral location of the tumor at the pons. Therefore, the patient was initially treated with prednisolone (0.5 mg/kg BW, twice daily, PO; Solondo^®^, Yuhan, Seoul, South Korea) and lomustine (60 mg/m^2^; CeeNU^®^, Bristol-Myers Squibb Co., Princeton, NJ, USA). Nine days after the commencement of chemotherapy, the patient experienced severe vomiting and hyporexia. Myelosuppression due to lomustine was not observed. The neurological signs did not improve; therefore, the chemotherapy was modified as follows: the lomustine was stopped, and a new regimen consisting of hydroxyurea (50 mg/kg BW, every other day, PO; Hydrin^®^, Korea United Pharm., Seoul, South Korea) plus imatinib (8 mg/kg BW, once daily, PO; Glima^®^, Boryung Pharmaceutical Co., Ltd, Seoul, South Korea) and prednisolone (0.5 mg/kg BW, twice daily, PO; Solondo^®^, Yuhan, Seoul, South Korea) was initiated. At 30 days after the initial chemotherapy, the slight leaning to the left improved, and the oculocephalic reflex was elicited. Therefore, a second MRI was performed to evaluate the therapeutic effectiveness through comparative analysis of tumor size. Although peritumoral edema was increased compared with that on the previous MRI finding, a decreased contrast-enhancing tumor size was observed on the second MRI (Figures 1E–H). At 107 days after commencement of therapy, the patient’s neurological signs were partially improved. However, owing to the occurrence of mild neutropenia (2.25 × 10^9^/L; reference range, 2.95–11.64 × 10^9^/L) and lymphopenia (0.93 × 10^9^/L; reference range, 1.05–5.10 × 10^9^/L), hydroxyurea was tapered to 30 mg/kg BW. At 213 days after the commencement of chemotherapy, the patient still showed a partial improvement in neurological signs, and there were no adverse effects of chemotherapy. A third MRI was performed to evaluate the therapeutic effectiveness on day 213. The third MRI showed decreased peritumoral edema compared with the findings of the previous scans (Figures 1I and J), and the contrast-enhancing tumor size had increased slightly (Figure 1K). Owing to the sustained clinical improvement without adverse effects of chemotherapy, from day 241 to day 310 after the initial chemotherapy, the dose of imatinib was tapered by 1 mg/kg BW every month until a dose of 5 mg/kg BW was reached. From day 310 after the initial chemotherapy, hydroxyurea (30 mg/kg BW, every other day), imatinib (5 mg/kg BW, once daily), and prednisolone (0.5 mg/kg BW, every third day) were administered as maintenance therapy. On day 569 after the initial chemotherapy, a fourth MRI scan was performed. Although peritumoral edema increased mildly in comparison with the findings of the third MRI scan, contrast enhancement was rarely observed (Figures 1M–P).

Additionally, ^18^F-fluorodeoxyglucose (^18^F-FDG)-positron emission tomography (PET) scan of the whole body, including the head, was performed immediately after the fourth MRI scan to determine the malignancy of the tumor and whether metastasis had occurred. ^18^F-FDG (5.92 MBq/kg BW) was administered intravenously into the right saphenous vein, followed by 5 mL of 0.9% normal saline for flushing of residual ^18^F-FDG. Low-dose CT images were acquired before PET scanning. Twenty-minute PET scans were obtained one hour after ^18^F-FDG injection. PET image analysis was performed using the OsiriX MD v10.0 (Pixmeo Sarl, Geneva, Switzerland). The regions of interest (ROIs) were drawn manually on the PET/CT fusion images. The metabolic activity of the ROIs was converted to a standardized uptake value (SUV) as follows: SUV = average tissue concentration of ^18^F-FDG (MBq/mL)/injected dose (MBq) per body weight (g). From a visual evaluation of the PET image, ^18^F-FDG uptake of the tumor was not remarkable compared with the surrounding region of the tumor (Figures 2A and B), and there was no evidence of metastatic lesion. The average and maximal SUVs of the tumor were 1.92 and 2.29, respectively. To evaluate metabolic activity more objectively, the tumor to normal tissue (T/N) ratio was calculated by dividing the maximal SUV of the tumor by the maximal SUV of the contralateral normal tissue using the dorsal plane, and a T/N ratio of 0.97 was recorded.

**Figure 2.**
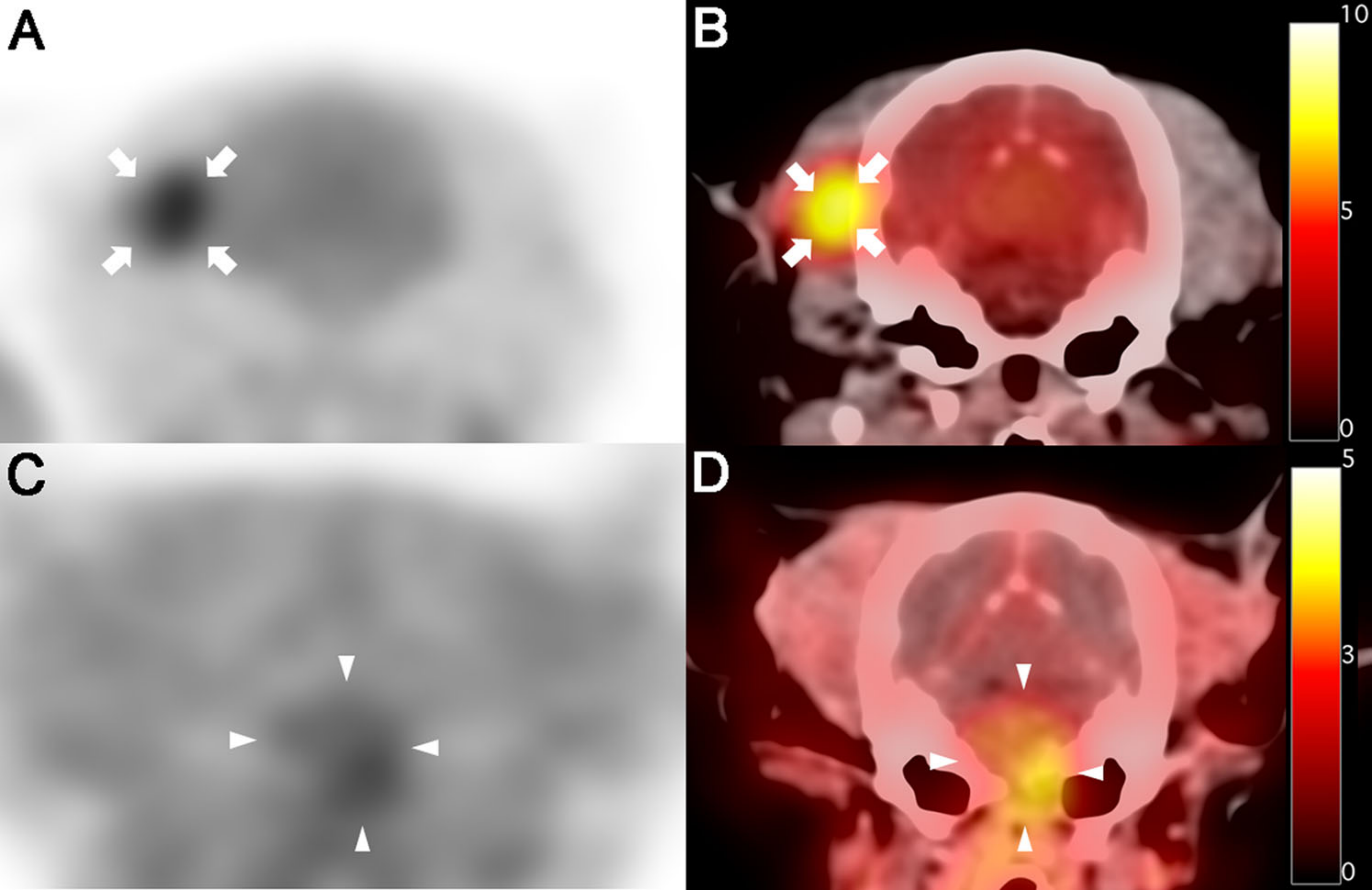
^18^F-fluorodeoxyglucose (^18^F-FDG) and ^18^F-fluorodopa (^18^F-FDOPA) positron emission tomography (PET)/CT findings in a dog with astrocytoma. (A, B)^18^F-FDG PET (A)and PET/CT fusion (B)images on day 569 after initial chemotherapy; no increase was observed in ^18^F-FDG uptake in the tumor lesion (pons) compared with the surrounding region. High ^18^F-FDG uptake was incidentally identified in the right temporal muscle (arrows). High ^18^F-FDG uptake is represented by black and yellow colors on PET and fusion images, respectively, while low ^18^F-FDG uptake is represented by white and red colors on PET and fusion images, respectively. (C, D)^18^F-FDOPA PET (C) and PET/CT fusion (**D**) images on day 1155 after initial chemotherapy; high ^18^F-FDOPA uptake was observed in the tumor lesion (arrowheads), and bone lysis was also observed in the background CT image. High ^18^F-FDOPA uptake is represented by black and yellow colors on PET and fusion images, respectively, while low ^18^F-FDOPA uptake is represented by white and red colors on PET and fusion images, respectively.

Based on the clinical signs and results of the fourth MRI and ^18^F-FDG PET, the previous doses of hydroxyurea, imatinib, and prednisolone were maintained. On day 1147 after the initial chemotherapy, the patient clinically deteriorated for the first time, manifesting circling, head turn, and recumbency. Therefore, the doses of hydroxyurea and prednisolone were increased to 50 mg/kg BW (every other day) and 1 mg/kg BW (twice daily), respectively. Despite the increased doses of hydroxyurea and prednisolone, the patient did not show signs of recovery; therefore, a fifth MRI and 3,4-dihydroxy-6-[^18^F]-fluoro-l-phenylalanine (^18^F-FDOPA) PET were performed to identify the tumor size at day 1155 after the initial chemotherapy. The fifth MRI was performed with a 1.5-Tesla unit MRI system (Signa Creator, GE Healthcare, Milwaukee, WI, USA). The tumor appeared much larger on the fifth MRI scan than on the previous scans (Figures 1Q–T). Furthermore, protrusion of the tumor into the nasopharynx was observed. ^18^F-FDOPA PET was performed following the same procedure as that adopted for ^18^F-FDG PET, except for the injection dose (2.47 MBq/kg BW) and interval time (10 min) from injection to scanning. On ^18^F-FDOPA PET/CT imaging, a high uptake of ^18^F-FDOPA was noted in the lesion, and protrusion of the tumor mass resulting from bone lysis was also identified (Figures 2C and D). The mean and maximal SUVs of the tumor were 1.59 and 2.29, respectively, and the T/N ratio was 2.22. Finally, based on the deterioration of clinical signs, MRI results, and ^18^F-FDOPA PET/CT results, the owner requested euthanasia, and the patient was euthanized on day 1155 after the initial treatment.

At necropsy, there was a well-defined mass lesion with hemorrhage in the pons (Figure 3). Histopathological examination revealed that the unencapsulated mass consisted of sheets of irregular interlacing fascicles of fibrillary spindle cells with severe diffuse microvascular proliferation and some mineralization (Figure 4A). Tumor cells surrounded the local extensive area of necrosis and were arranged in relation to each other in a manner similar to a palisade or slats in a picket fence. Vascular proliferations were observed, including an increased number of congested branched vessels lined by a single layer of hypertrophic endothelium (Figure 4B). Tumor cells exhibited abundant eosinophilic cytoplasm, with round to oval basophilic nuclei that appeared similar to astrocytes with low mitotic activity and high nuclear pleomorphism. Based on these findings and the World Health Organization classification, a definitive diagnosis of low-grade glioma (astrocytoma) was made (Villa et al. 2018).

**Figure 3.**
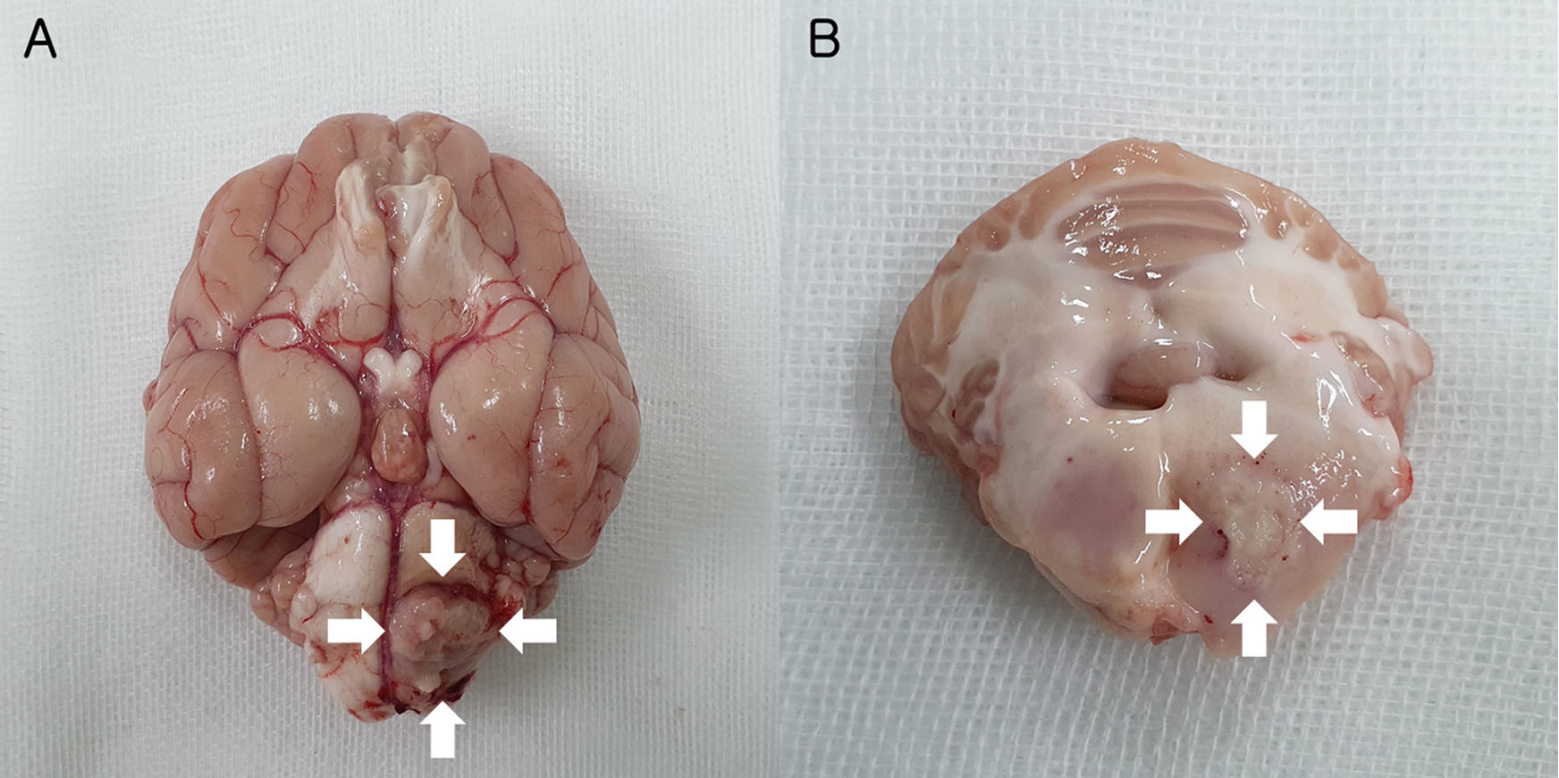
Necropsy findings of an astrocytoma in an 8-year-old neutered male Yorkshire Terrier dog. (A)Photograph showing a mass lesion (arrows) derived from the parenchyma. The mass protruded through the meninges. (B)Well-defined mass (arrows) with hemorrhage in the left pons.

**Figure 4.**
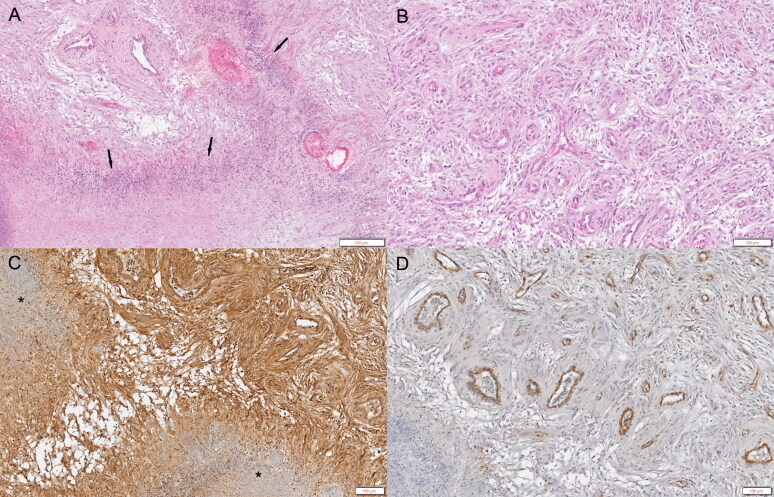
Histopathological and immunohistochemical evaluation of an astrocytoma in an 8-year-old neutered male Yorkshire Terrier dog. (A)Unencapsulated mass comprising sheets of fibrillary spindle cells with severe diffuse microvascular proliferation. Tumor cells are lined up next to each other and are surrounding the local extensive area of necrosis (arrows). Hematoxylin and eosin (H&E) stain. Scale bar = 200 µm. (B)Vascular proliferations with increased numbers of congested, branching vessels lined by a single layer of hypertrophied endothelium. H&E stain. Scale bar = 100 µm. (C)Widespread distribution of L-type amino acid transporter 1 (LAT1) positive staining surrounding a local extensive area of necrosis (asterisks). LAT1 immunohistochemistry. Scale bar = 100 µm. (D)Platelet-derived growth factor receptor (PDGFR)-β positive staining observed in the vascular endothelium. PDGFR-β immunohistochemistry. Scale bar = 100 µm.

Both L-type amino acid transporter 1 (LAT1) and platelet-derived growth factor receptor (PDGFR)-β antibodies were used for immunohistochemistry (IHC) analysis. Anti-LAT1 and anti-PDGFR-β antibodies were purchased from Abcam (Cambridge, UK). The VECTASTAIN elite ABC-HRP kit was obtained from Vector Laboratories (Burlingame, CA, USA). LAT1 IHC staining revealed that the tumor mass was strongly positive in the cytoplasm, plasma membranes, and vascular endothelium surrounding the local extensive area of necrosis (Figure 4C). In addition, PDGFR-β IHC staining showed selectively positive staining in the vascular endothelium (Figure 4D).

In human oncology, ^18^F-FDG PET has been used to distinguish benign from malignant tumors (Delbeke et al. 1998; Nakamoto et al. 2000) and to confirm metastases (Weder et al. 1998) and residual lesions following surgery and radiotherapy (Bogsrud et al. 2010). Although there are many reports of brain tumors in humans, to the best of our knowledge, only a single case of ^18^F-FDG PET findings of the brain tumor (histiocytic sarcoma) has been reported in the dog species (Kang et al. 2009), and there are no reports of ^18^F-FDOPA PET characteristics in canine brain tumors. Imaging of brain tumors with ^18^F-FDG PET has been limited due to the characteristically high physiological glucose uptake by normal tissue (Olivero et al. 1995; Barker et al. 1997). Therefore, the detection of low-grade tumors and recurrent tumors that have lesions with mildly increased uptake of glucose is difficult to achieve with ^18^F-FDG PET (Olivero et al. 1995; Barker et al. 1997). For this reason, PET tracers of the amino acid class, which possess the characteristics of low uptake in normal brain tissue but high uptake in tumor lesions, have been used for imaging brain tumors (Chen et al. 2006; Tripathi et al. 2009; Wardak et al. 2014). Among the amino acid analog tracers, ^18^F-FDOPA is one of the representative amino acid PET tracers for human brain tumors (Chen et al. 2006; Wardak et al. 2014). ^18^F-FDOPA PET (96%) is much better than ^18^F-FDG PET (61%) for the visualization of human gliomas (Chen et al. 2006). In the present case, the tumor lesion was not detected by ^18^F-FDG PET but was identified by ^18^F-FDOPA PET based on visual analysis, revealing the superiority of ^18^F-FDOPA PET over ^18^F-FDG PET even in a single case. The SUVmax of human low-grade glioma in ^18^F-FDG PET and ^18^F-FDOPA PET was 2.48 ± 0.85 and 3.07 ± 1.65, respectively (Chen et al. 2006), and the T/N ratios of human low-grade glioma in ^18^F-FDG PET and ^18^F-FDOPA PET were 1.03 ± 0.64 and 2.3 ± 0.51, respectively (Tripathi et al. 2009). In the present case, canine low-grade glioma in ^18^F-FDG PET and ^18^F-FDOPA PET had the same SUVmax (2.29), and the T/N ratios of canine low-grade glioma in ^18^F-FDG PET and ^18^F-FDOPA PET were 0.97 and 2.22, respectively. Although both the SUVmax values of canine low-grade glioma were lower than those of humans, the T/N ratio, which is an objectively quantified value, was similar in humans and dogs.

The oculocephalic reflex (physiologic nystagmus) requires the vestibular system and its connected components to be normal. However, when bilateral defects in the vestibular system and its related connections occur, physiologic nystagmus is not elicited. Therefore, the recovery of physiologic nystagmus, which was not initially observed, was due to the improvement of the lesion in the brainstem after chemotherapy with prednisolone was initiated.

Several treatment methods, including surgery, radiotherapy, symptomatic management, chemotherapy, and combined therapies, have been used in canine brain tumors (Hu et al. 2015). The longest median survival time among the treatments was observed with radiotherapy (351 days) (Hu et al. 2015). The median survival times for symptomatic and surgical treatment have been reported to be 65 and 312 days, respectively (Hu et al. 2015). Because radiotherapy and surgery have shown good prognosis, they have been commonly opted as the first choice treatment. However, surgery and radiation therapy cannot be applied in some dogs due to tumor location or financial constraints. Chemotherapy with symptomatic management could be used as an alternative for dogs with a limited chance of surgery or radiotherapy. There has been a case report of survival of up to 14 months after chemotherapy with hydroxyurea in meningioma (Tamura et al. 2007). In the present case, the dog was treated with combination therapy of hydroxyurea plus imatinib and survived for 38 months (1155 days); this case report documents the longest survival of a dog with a brain tumor treated with chemotherapy alone.

Hydroxyurea is an oral antineoplastic drug that induces tumor cell apoptosis (Schrell et al. 1997) and selectively inhibits ribonucleotide reductase, thereby preventing DNA synthesis (Elford 1968). Although there is controversy over its efficacy in brain tumors, hydroxyurea is a representative chemotherapeutic agent that has been applied frequently for the treatment of meningiomas in human and veterinary medicine (Newton et al. 2000; Forterre et al. 2006; 2007; Tamura et al. 2007; Jung et al. 2014). In veterinary medicine, some studies have reported that hydroxyurea is effective in the treatment of meningiomas (Tamura et al. 2007; Cho et al. 2018). In human malignant glioma, nitrosourea-based chemotherapeutic regimens and temozolomide are generally administered (Kaba and Kyritsis 1997; Stewart 2002; Stupp et al. 2005). Hydroxyurea is also used in the treatment of human gliomas; however, it is used as an adjuvant drug to a nitrosourea-based protocol or as an alternative drug to progressive pretreated glioblastoma multiforme refractory to temozolomide (Kaba and Kyritsis 1997; Dresemann et al. 2010). In the current case, because of the severe adverse effects of lomustine, despite a glioma being strongly suspected based on MRI findings, hydroxyurea was alternatively used.

Tyrosine kinases are a large family of intracellular and membrane-bound enzymes that transfer a phosphate group to the tyrosine residue of other proteins. Tyrosine kinases trigger cell proliferation, differentiation, migration, and metabolic changes via numerous signaling cascades (Schlessinger and Ullrich 1992). Dysregulation (mutation) of tyrosine kinases induces uncontrolled cell proliferation and differentiation. In veterinary medicine, there is limited information on the clinical efficacy of tyrosine kinase inhibitors. Imatinib is a selective inhibitor of tyrosine kinase that targets c-Kit, BCR-Abl, and PDGFR. It has been used to treat gastrointestinal stromal tumors, chronic myelogenous leukemia, and various other malignant tumors in humans (Druker at al. 2001; Joensuu et al. 2001; Demetri et al. 2002; van Oosterom et al. 2002). The application of imatinib as a single agent has limited antitumor activity in human malignant gliomas (Wen et al. 2006). Despite the controversy over the efficacy of imatinib, some pilot studies have reported that the combination therapy of hydroxyurea and imatinib is tolerable and efficacious for glioblastoma multiforme (Dresemann 2005; Reardon et al. 2005). In veterinary medicine, imatinib has been used in mast cell tumors, gastrointestinal stromal tumors, and feline vaccine-associated sarcoma (Pryer et al. 2003; Katayama et al. 2004; Kobayashi et al. 2013). There have been no reports of cases of glioma treated with imatinib; however, the combination therapy of hydroxyurea and imatinib has shown good efficacy for meningiomas in veterinary medicine (Jung et al. 2014). In the current study, positive staining for PDGFR-β, targeted by imatinib, was observed in the vascular endothelium. Although combination therapy with hydroxyurea and imatinib was used, imatinib was initially associated with an improvement in neurological clinical signs.

Unlike monotherapy, the combination of hydroxyurea and imatinib was associated with the patient’s neurological improvement. The exact mechanism of hydroxyurea plus imatinib is unknown; however, several mechanisms have been proposed. Imatinib can reduce the interstitial pressure of tumors, which could lead to enhanced hydroxyurea delivery by increased capillary-to-interstitium transport (Pietras et al. 2001). Imatinib also suppresses the function of the multidrug transporters (ABCG2) responsible for the transportation of xenobiotics (Houghton et al. 2004). Therefore, this combination regimen modulates the transportation of both drugs at the tumor cell membrane and the blood-brain barrier and prolongs the concentration of both drugs in the central nervous system (Pietras et al. 2001; Houghton et al. 2004).

Glucocorticoids have generally been used to reduce edema in patients with brain tumors for early supportive care. Although the exact mechanisms that reduce tumor-surrounding edema are only partially understood, in a preclinical study using rat glioma models, dexamethasone treatment reduced the vascular permeability within the tumor (Guerin et al. 1992). Therefore, the recovery of neurological signs in the present case was due to not only chemotherapy but also prednisolone by reducing cerebral edema.

LAT1 is a neutral or aromatic amino acid transport system associated with the permeation of amino acids, such as tryptophan, phenylalanine, leucine, isoleucine, methionine, valine, and amino acid analogs, such as levodopa (L-DOPA) (Kanai et al. 1998; Yanagida et al. 2001). Its normal expression is restricted to the blood-brain barrier, spleen, placenta, testis, and colon (Kanai et al. 1998; Matsuo et al. 2000); it plays a crucial role in the uptake and efflux of amino acids through blood-tissue barriers (Kanai et al. 1998; Kageyama et al. 2000; Yanagida et al. 2001). LAT1 has also been reported to be expressed in many tumor cell lines requiring many amino acids for proliferation, and its expression is lower in normal tissues (Wolf et al. 1996; Kanai et al. 1998; Yanagida et al. 2001; Kim et al. 2004; Nawashiro et al. 2006). The overexpression of LAT1 has been found to be specific for metastases as compared to normal tissue (Ichinoe et al. 2015; Papin-Michault et al. 2016; Higuchi et al. 2019), and one study showed that LAT1 plays an important role in the uptake of amino acids in brain metastases (Papin-Michault et al. 2016). ^18^F-FDOPA is radiolabeled L-DOPA with the positron emitter isotope ^18^F, and it also enters into the same L-DOPA metabolic pathway (Luxen et al. 1992). For this reason, ^18^F-FDOPA has been used as a PET imaging agent to identify enhanced amino acid transport and protein synthesis in human patients with tumors (Chen et al. 2006; Wardak et al. 2014). The degree of LAT1 expression has a strong correlation with the uptake of ^18^F-FDOPA in biopsy samples of patients (Youland et al. 2013). The immunoreactivity for LAT1 was previously identified in biopsy samples from a glioma (Haining et al. 2012), indicating that LAT1 might be a molecular target for diagnostic imaging (Nawashiro et al. 2006). In the present case, LAT1 IHC staining revealed that the tumor mass was strongly positive for LAT1 in the cytoplasm, plasma membranes, and vascular endothelium surrounding the local extensive area of necrosis. Because it is a single case, the correlation between the expression levels of LAT1 and ^18^F-FDOPA was not confirmed. However, the accumulation of more cases could allow the use of ^18^F-FDOPA PET to evaluate the degree of malignancy and metastasis of brain tumors.

To the best of our knowledge, this is the first reported case of ^18^F-FDG and ^18^F-FDOPA PET results in a dog with glioma. In the present case, because the tumor was not detected by ^18^F-FDG, but by ^18^F-FDOPA PET, the latter can be used for a better identification of low-grade gliomas. This data may provide valuable diagnostic information for understanding canine gliomas in veterinary medicine, and further studies are needed to establish diagnostic and staging criteria for canine gliomas. Furthermore, there have been no previous reports of canine gliomas treated only with a chemotherapy combination of hydroxyurea and imatinib. Our findings suggest that chemotherapy with hydroxyurea and imatinib may be considered as an alternative treatment for canine gliomas when surgery and radiotherapy are limited for various reasons.
